# Exploring the Causal Relationships and Mediating Factors Between Mental Disorders and Hypertension: A Multivariable Mendelian Randomization Study

**DOI:** 10.5334/gh.1483

**Published:** 2025-10-14

**Authors:** Kunyan Li, Chun Yin, Hao Yang, Zhichun Gao, Wang Dong, Jun Jin

**Affiliations:** 1 Department of Cardiology, Xinqiao Hospital, Army Medical University, Chongqing, China; 2 Department of Cardiology, the 902nd Hospital of PLA Joint Service Support Force, Bengbu, China

**Keywords:** mental disorders, depression, hypertension, Multivariable Mendelian randomization, mediation analysis

## Abstract

**Background:**

Previous studies have demonstrated a correlation between mental disorders and hypertension. However, the direction of this association and the specific risk factors that mediate the causal effects remain unknown. The present study aimed to investigate the causal relationship between mental disorders and hypertension, as well as identify the risk factors that mediate it.

**Methods:**

We used univariate Mendelian randomization (UVMR) and multivariate Mendelian randomization (MVMR) to evaluate the causal relationship between depression, anxiety, or panic attacks and hypertension using the summarized statistics from eleven extensive genome-wide association studies in European populations. Furthermore, MVMR was used to evaluate seven potential mediators of this association and calculate their mediated proportions. The robustness of our findings was evaluated using sensitivity analyses.

**Results:**

UVMR analysis revealed that genetically predicted higher risk of depression (OR: 1.140, 95%CI: [1.075, 1.210], p < 0.001), anxiety (OR: 2.679, 95%CI: [1.328, 5.408], p < 0.01), and panic attacks (OR: 1.054, 95%CI: [1.016, 1.092], p < 0.01) were associated with increased risk of hypertension. Higher risk of hypertension was also associated with higher risk of depression (OR: 1.101, 95%CI: [1.009, 1.202], p < 0.05). Of seven candidate mediators, two met the screening criteria and were included in the mediation MR analyses. The MVMR analysis revealed that even after adjusting for depression, there was a persistent causal relationship between type 2 diabetes and hypertension (OR: 1.010, 95%CI: [1.005, 1.015], p < 0.001). Similarly, the causal relationship between smoking and hypertension remained significant after accounting for depression (OR: 1.037, 95%CI: [1.015, 1.060], p < 0.001). Mediation analyses indicated that diabetes and smoking have mediation effects of 8.71% and 5.79% between depression and hypertension, with mediation proportions of 41.7% and 27.7%, respectively.

**Conclusion:**

This study provided compelling evidence supporting a bidirectional phenotypic association between depression and hypertension, while highlighting diabetes and smoking as significant mediators in the association’s pathway to hypertension development.

## Introduction

Hypertension has emerged as a significant menace to global public health, primarily due to its detrimental impact on the heart, blood vessels, and artery walls (1,2). Epidemiological reports indicate that approximately one-third of the global population is affected by hypertension (3). This widespread prevalence underscores the gravity of the issue. When left undiagnosed and untreated in its early stages, hypertension can trigger a cascade of severe complications, including strokes and coronary artery diseases (4,5). As the World Health Organization (WHO) has pointed out, hypertension directly accounts for 54% of all stroke cases and 47% of ischemic heart disease instances (6). These statistics highlight the critical role of early detection and intervention in hypertension management. A multitude of risk factors have been associated with hypertension. Among them are type 2 diabetes mellitus (T2DM), smoking, elevated blood urea nitrogen (BUN) levels, a high body mass index (BMI), abnormal triglycerides (TG) levels, low levels of high-density lipoprotein (HDL), and renal failure (7). Moreover, recent research has suggested that psychological and mental disorders can also heighten an individual’s susceptibility to hypertension (8). Understanding these risk factors is essential for formulating effective prevention and treatment strategies.

Depression, panic attacks, and anxiety are prevalent symptoms of mental disorders. An epidemiological survey indicated that in 2007, depression emerged as a widespread mental health condition, impacting more than 264 million people globally (9). It not only ranks as the primary cause of disability but also imposes a substantial burden on public health systems across the world (10). Although some observational research has addressed the association between depression and hypertension, the exact nature of this relationship remains elusive. Previous studies examining the link between the two have yielded inconsistent results. Some research has shown a positive correlation (11,12,13), others a negative one (14,15), and yet some have found no significant effect (16). Moreover, only a limited number of studies have explored the possibility of a bidirectional relationship between depression and hypertension. One prospective cohort study did investigate this bidirectional relationship. It discovered that elevated blood pressure is associated with a decreased risk of incident case-level depressive symptoms (OR = 0.77, 95%CI: 0.60, 0.99), while depressive symptoms are linked to an increased risk of hypertension (OR = 1.29, 95%CI: 1.10, 1.50) (17). Given the inconclusive findings from previous research and the scarcity of investigations into the bidirectional association, well-powered studies are needed to re-examine the relationship between depression and hypertension.

Mendelian randomization (MR) analysis, a sophisticated statistical method, is employed to establish the causal relationship between an exposure and an outcome. By leveraging genetic variants associated with the exposure as instrumental variables (IVs), it allows for a more accurate evaluation of the relationship between the exposure and the outcome. One of the key advantages of MR analysis lies in its robustness against confounding factors. Unlike traditional observational or cross-sectional studies, MR benefits from the random allocation of genetic alleles during gametogenesis. The randomness ensures that the genetic variants used as IVs are less likely to be influenced by external confounding variables, providing a more reliable causal inference. Additionally, since genotypes are determined before the onset of disease, MR analysis effectively mitigates the issue of reverse causation bias. MR analysis has been widely applied in various scenarios, including investigations into the causal associations between mental disorder and hypertension. Previous MR studies have provided some insights; for instance, they have suggested that depression is a causal risk factor for hypertension, while the reverse relationship may not hold true (18,19). However, another MR study failed to establish a causal relationship between blood pressure and anxiety or depressive symptoms (20).

Multivariable Mendelian randomization (MVMR), an extension of the MR method, offers a more comprehensive approach. It enables researchers to explore the independent or combined effects of multiple exposures on an outcome by utilizing genetic variations associated with them. This not only aids in understanding complex causal networks but also allows for the investigation of the potential mediating effects of intermediate characteristics between the exposure and the outcome (21). Despite the existing research on the potential causal relationship between depression and hypertension, the mediating impact of depression on hypertension remains poorly understood.

In this study, we conducted UVMR and MVMR analyses to explore the causality between mental disorders and hypertension. Moreover, we aimed to assess the potential mediating roles of T2DM and smoking in this pathway from depression to hypertension.

## Methods

### Study design

**Figure 1:**
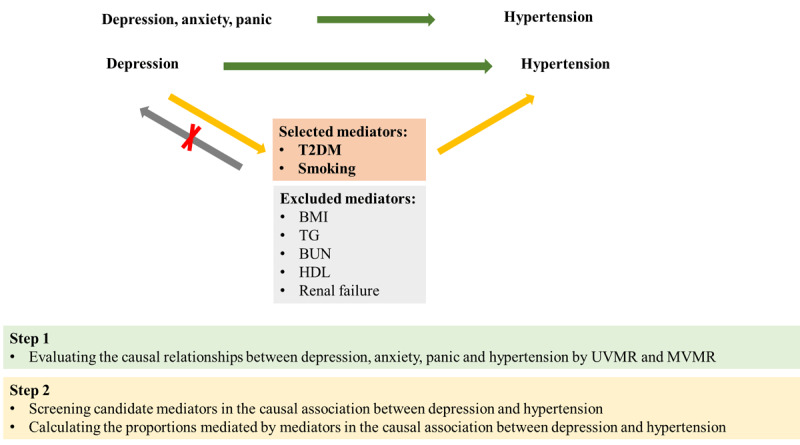
Overview of the study design. Step 1: we used UVMR and MVMR to evaluate the causal relationships between depression, anxiety, panic, and hypertension. Step 2: we screened candidate mediators in the causal association between depression and hypertension, and then we calculated their mediating effects using MVMR. T2DM, type 2 diabetes mellitus; BMI, body mass index; TG, triglycerides; BUN, blood urea nitrogen; HDL, high density lipoprotein.

We conducted UVMR and MVMR analysis using data from GWAS to determine the causal relationship between mental disorders and hypertension based on the latest (STROBE-MR) guidelines (22). The study design flowchart is shown in Figure 1. This study comprises two main steps. In the first step, we employed UVMR to investigate potential causal links between mental disorders and hypertension. Additionally, MVMR was used to further assess the direct effect of depression, anxiety, and panic attacks on hypertension. Reverse MR analyses were then conducted to determine the causal effect of hypertension on various exposures. In the second step, we screened candidate mediators in the causal association between depression and hypertension, followed by calculation of their mediating effects using MVMR. The following three assumptions are satisfied by this study: (1) the IVs exhibit a substantial correlation with the exposure phenotype; (2) the association between IVs and outcome must be independent of confounding factors; and (3) the IVs solely impact outcomes through the exposure factor.

### Data sources

We obtained summary-level association results from the largest available genome-wide association studies (GWAS) conducted for depression, anxiety, panic attacks, T2DM, smoking, BUN, BMI, triglycerides, HDL, acute renal failure, and hypertension, with a primary focus on individuals of European ancestry. The phenotype for hypertension is ICD10: I10, referring to ‘Essential (primary) hypertension’, which indicates that the participant has been diagnosed with ‘Essential hypertension’. The phenotype for anxiety is ICD10: F41.9, referring to ‘Anxiety disorder, unspecified’, which indicates that the participant has been diagnosed with ‘unspecified anxiety disorder’. The phenotype for renal failure is ICD10: N17.9, referring to ‘Acute renal failure, unspecified’, which indicates that the participant has been diagnosed with ‘unspecified acute renal failure’. The phenotype for panic refers to ‘Mental health problems ever diagnosed by a professional: Panic attacks’. The phenotype for smoking refers to ‘pack years of smoking’. The phenotype for depression refers to ‘broad depression’.

These GWAS datasets are obtained from the IEU GWAS project (https://gwas.mrcieu.ac.uk/datasets/) and the GWAS catalog website (https://www.ebi.ac.uk/gwas/). The study population consisted of 208,811 controls and 113,769 cases for depression (23), 111,204 controls and 6,518 cases for panic attacks, 461,487 controls and 1,523 cases for anxiety, 5,794 controls and 6,377 cases for T2DM (24), 408,652 controls and 54,358 cases for hypertension, 461,595 controls and 1,415 cases for acute renal failure, 142,387 participants for smoking, 99,998 participants for BMI, 441,016 participants for TG (25), 62,403 participants for BUN (26), as well as 88,329 participants for HDL (27). Details on these data sources are presented in Table 1. All the original studies in this study obtained ethical approval and informed consent.

**Table 1:** Summary of GWAS data used in the MR analyses.


PHENOTYPE	NO. OF PARTICIPANTS	nSNPs	UNIT	ANCESTRY	CONSORTIUM/COHORT	AUTHOR	YEAR OF PUBLICATION	PMID

Depression	322580	7624934	logOR	European	–	Howard DM	2018	29662059

Anxiety	463010	9851867	SD	European	MRC–IEU	Ben Elsworth	2018	–

Panic	117722	12509516	–	European	–	Neale lab	2018	–

Hypertension	463010	9851867	SD	European	MRC–IEU	Ben Elsworth	2018	–

T2DM	69033	2905211	logOR	European	–	Morris AP	2012	22885922

Smoking	142387	9851867	SD	European	MRC–IEU	Ben Elsworth	2018	–

BMI	99998	7191606	–	European	Within family GWAS consortium	Howe LJ	2022	–

TG	441016	12321875	–	European	UK Biobank	Richardson Tom	2020	32203549

BUN	62403	–	–	European	–	Dennis JK	2021	33441150

HDL	88329	–	–	European	–	Davyson E	2023	36764567

Renal failure	463010	9851867	SD	European	MRC–IEU	Ben Elsworth	2018	–


T2DM: type 2 diabetes mellitus, BMI: body mass index, TG: triglycerides, BUN: blood urea nitrogen, HDL: high density lipoprotein.

### Instrumental variable selection

For the MR analysis, IVs for exposure were selected based on the following procedure. Initially, single nucleotide polymorphisms (SNPs) strongly associated with exposure were selected, with a significance threshold of p < 5 × 10^–8^. Given the limited number of IVs for anxiety and panic attacks, we relaxed our significance threshold to 5 × 10^–5^ in order to avoid potential errors arising from a restricted SNP pool size. Subsequently, it is necessary to eliminate linkage disequilibrium and ensure the independence of SNPs, because linkage disequilibrium leads to bias in MR analysis. The parameters are set as follows: r2 = 0.001 and kb = 10,000. Furthermore, F statistics were used to evaluate the strength of the IVs to avoid weak-tool bias. If F < 10, indicating weak correlation, the corresponding instrumental variable is excluded.

### MR analysis

#### UVMR analysis

We performed UVMR analyses, which harmonized the SNPs in the datasets and removed all palindromic SNPs from the analysis, to determine the causal effect of exposure on outcome. The main method for causal estimation was the Wald method when only one SNP tool was available. When multiple valid instrumental variables were available, five methods were employed: inverse variance weighting (IVW), MR Egger, weighted median, simple mode, and weighted mode. Among them, IVW was the primary method, while the others served as supplementary approaches. The IVW method meta-analyzed SNP-specific Wald estimates using random effects to obtain the final estimate of the causal effect, which was reported as a beta value with a standard error for continuous outcomes and an odds ratio with a 95% confidence interval for binary outcomes. A two-sided *P* value <0.05 was considered nominally significant.

#### MVMR analysis

We performed MVMR to estimate the direct effect of depression, anxiety, or panic attacks on hypertension and mutually adjusted them to determine which exposure was causally associated with hypertension independently of the other two exposures. We primarily conducted the MV-IVW method, supplemented by several sensitivity analyses (namely MV-Egger, MV-LASSO, and MV-Median) to assess the robustness of the results. A causal association was considered if the MV-IVW estimate agreed with at least one sensitivity analysis for directionality and statistical significance but showed no evidence of pleiotropy (P > 0.05). To mitigate the issue of multiple comparisons, we employed the Bonferroni method in the MVMR analysis with a significance threshold of P < 0.016 (0.05/3 = 0.016).

#### Mediation MR Analyses

Based on literature reviews, we selected seven candidate mediators associated with modifiable hypertension risk factors, which could potentially lie on the causal pathways linking depression to hypertension. These mediators were chosen based on the availability of genetic instruments derived from GWASs, including BUN, BMI, TG, T2DM, HDL, smoking, and acute renal failure. Subsequently, we screened these candidate mediators according to the following criteria: (1) There exists a causal association between depression and the mediator, and the effect of depression on the mediator should be unidirectional, because the validity of the mediation analyses may be affected if bidirectionality exists between them; (2) the causal association consistently exists between the mediator and hypertension regardless of adjustment for depression; and (3) the association between depression and the mediator should align with the association between the mediator and hypertension. The detailed process for selecting mediators is illustrated in Figure 1. Ultimately, two risk factors met all criteria and were included in subsequent mediation analyses.

We conducted MVMR analysis to evaluate their mediating effects on the causal association between depression and hypertension. Firstly, UVMR was used to estimate the genetically determined effect of depression on hypertension (C). Then, UVMR was applied again to estimate the genetically determined effect of depression on each mediating factor individually (A). Finally, MVMR was conducted to estimate the causal effect of each mediating factor (B) independently on hypertension, while adjusting for depression (C’). The indirect effect can be estimated by subtracting the direct effect (C’) from the total effect (C) (28). The proportion of the total effect of depression on hypertension mediated by T2DM and smoking is determined by dividing the indirect effect (C-C’) by the total effect (C). We employed the Delta method, using effect estimates derived from MVMR analysis to obtain the standard error of the mediation proportion and subsequently calculate its confidence interval.

#### MR sensitivity analysis

To ensure the reliability and robustness of the causality assessment results, sensitivity analyses were performed. The Cochrane Q statistic was used to evaluate heterogeneity between instrumental variables. When the p-value exceeded 0.05, indicating the absence of heterogeneity, the fixed-effects inverse-variance weighting method was chosen as the primary approach. In contrast, a random-effects model was used when heterogeneity was detected. The MR-Egger intercept test and MR-PRESSO global test were employed to assess horizontal pleiotropy among the selected instrumental variables. The MR-PRESSO outlier test was used to identify potential outliers. If an outlier SNP was found (p < 0.05), the causal effects were re-estimated using the remaining SNPs after removing the outliers. A leave-one-out sensitivity analysis was performed to assess whether the results were driven by individual SNPs. To directly assess the presence of heterogeneity, funnel plots were created. In the MVMR analysis, pleiotropy was tested using the MR Egger intercept, and heterogeneity between instruments was assessed using the Q’ heterogeneity statistic.

All MR analyses were performed using the ‘TwoSampleMR’, ‘MVMR’, ‘MR-PRESSO’, and ‘RMediation’ packages in R version 4.3.3 (R Foundation for Statistical Computing, Vienna, Austria).

## Results

### SNP selection

Due to being palindromic with intermediate allele frequencies, rs587510 was removed from the panic attacks. The MR-PRESSO outlier test showed that rs116492571, rs12911514, and rs34324971 were identified as potential outliers and then removed from the panic attacks. Consequently, we obtained 14 SNPs for depression, 18 SNPs for anxiety, and 96 SNPs for panic attacks. The F-statistics of all selected SNPs were greater than 10, indicating no weak instrument bias. Details of the selected IVs are shown in Supplementary Tables S1–3. As a result, no proxy SNPs were found.

### Total and direct effects of depression, anxiety, or panic on hypertension

In the UVMR analysis, the three datasets showed that genetically predicted higher risk of depression (OR: 1.140, 95%CI: [1.075, 1.210], p < 0.001), higher risk of anxiety (OR: 2.679, 95%CI: [1.328, 5.408], p < 0.01), higher risk of panic attacks (OR: 1.054, 95%CI: [1.016, 1.092], p < 0.01), are associated with higher risk of hypertension (Table S4, Figure S1). Higher risk of hypertension are associated with higher risk of depression (OR: 1.101, 95%CI: [1.009, 1.202], p < 0.05), while hypertension has no reverse causal relationship with anxiety (OR: 1.006, 95%CI: [0.999, 1.014], p > 0.05) and panic attacks (OR: 1.018, 95%CI: [0.966, 1.072], p > 0.05) (Table S5). In the UVMR analysis, the MR results of depression are stable for at least one sensitivity analysis. These genetic instrumental variables for depression, anxiety, and panic attacks show no heterogeneity (Table S6). Furthermore, leave-one-out analysis revealed that no SNP significantly influenced the results (Figure S2), and funnel plots displayed symmetrical distributions (Figure S3). These genetic instrumental variables for hypertension do not have horizontal pleiotropy (Table S7).

**Figure 2:**
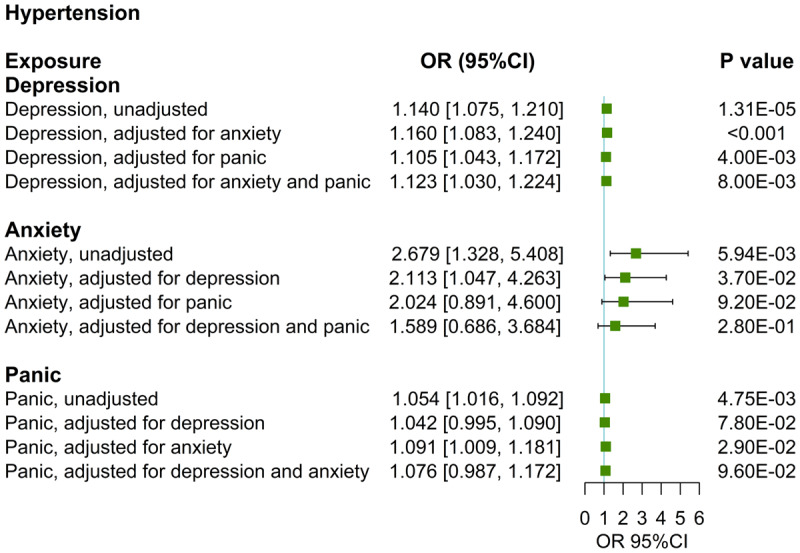
UVMR and MVMR estimates of the causal associations of depression, anxiety, and panic with hypertension.

In the MVMR analysis, the causal relationship between depression and hypertension persists after adjusting for anxiety (OR: 1.160, 95%CI: [1.083, 1.240], p < 0.001), panic attacks (OR: 1.105, 95%CI: [1.043, 1.172], p < 0.01) or both (OR: 1.123, 95%CI: [1.030, 1.224], p < 0.01) (Table 2, Figure 2). The causal relationship between anxiety and hypertension loses statistical significance after adjusting for depression (OR: 2.113, 95%CI: [1.047, 4.263], p > 0.016), panic attacks (OR: 2.024, 95%CI: [0.981, 4.600], p > 0.016) or both (OR: 1.589, 95%CI: [0.686, 3.684], p > 0.016) (Table 3, Figure 2). The causal relationship between panic attacks and hypertension loses statistical significance after adjusting for anxiety (OR: 1.091, 95%CI: [1.009, 1.181], p > 0.016), depression (OR: 1.042, 95%CI: [0.995, 1.090], p > 0.05) or both (OR: 1.076, 95%CI: [0.987, 1.172], p > 0.016) (Table 3, Figure 2). All directions and statistical significances of the IVW results in MVMR are consistent with the results of the MV-Egger analysis, and the MVMR Egger intercept analysis shows that there is no pleiotropy in the instrumental variables (Table 2).

**Table 2:** MVMR estimating the associations of depression, panic, and anxiety with hypertension.


METHOD	EXPOSURE	β (95%CI)	OR (95%CI)	P VALUE	NO OF SNPs	MVMR HETEROGENEITY TEST (IVW/EGGER)		MVMR DIRECTIONAL PLEIOTROPY TEST		
	
						Q	P	EGGER INTERCEPT	SE	P

Hypertension										

Models with mutual adjustment for depression, anxiety and panic										

MV-IVM	depression	0.116 [0.03, 0.202]	1.123 [1.030, 1.224]	0.008	46	71.5173	0.0041			

	anxiety	0.463 [–0.377, 1.304]	1.589 [0.686, 3.684]	0.280						

	panic	0.073 [–0.013, 0.159]	1.076 [0.987, 1.172]	0.096						

MV-Egger	depression	0.164 [0.052, 0.275]	1.178 [1.053, 1.317]	0.004		68.7607	0.0057	0	0	0.194

	anxiety	0.596 [–0.262, 1.454]	1.815 [0.770, 4.280]	0.173						

	panic	0.079 [–0.007, 0.165]	1.082 [0.993, 1.179]	0.070						

MV-LASSO	depression	0.146 [0.067, 0.225]	1.157 [1.069, 1.252]	<0.001						

	anxiety	0.543 [–0.19, 1.276]	1.721 [0.827, 3.582]	0.147						

	panic	0.102 [0.024, 0.179]	1.107 [1.024, 1.196]	0.010						

MV-median	depression	0.128 [0.026, 0.23]	1.137 [1.026, 1.259]	0.014						

	anxiety	0.317 [–0.649, 1.284]	1.373 [0.523, 3.611]	0.520						

	panic	0.084 [–0.023, 0.19]	1.088 [0.977, 1.209]	0.124						

Models with mutual adjustment for depression and anxiety										

MV-IVM	depression	0.148 [0.08, 0.215]	1.160 [1.083, 1.240]	<0.001	27	32.4864	0.1444			

	anxiety	0.748 [0.046, 1.45]	2.113 [1.047, 4.263]	0.037						

MV-Egger	depression	0.178 [0.075, 0.282]	1.195 [1.078, 1.326]	0.001		31.6913	0.1348	0	0	0.438

	anxiety	0.858 [0.098, 1.618]	2.358 [1.103, 5.043]	0.027						

MV-LASSO	depression	0.148 [0.08, 0.215]	1.160 [1.083, 1.240]	<0.001						

	anxiety	0.748 [0.046, 1.45]	2.113 [1.047, 4.263]	0.037						

MV-median	depression	0.165 [0.073, 0.257]	1.179 [1.076, 1.293]	<0.001						

	anxiety	0.414 [–0.496, 1.324]	1.513 [0.609, 3.758]	0.373						

Models with mutual adjustment for depression and panic										

MV-IVM	depression	0.1 [0.042, 0.159]	1.105 [1.043, 1.172]	0.004	93	125.3549	9.90E-03			

	panic	0.041 [–0.005, 0.086]	1.042 [0.995, 1.090]	0.078						

MV-Egger	depression	0.113 [0.036, 0.19]	1.120 [1.037, 1.209]	0.004		125.0206	8.60E-03	0	0	0.624

	panic	0.043 [–0.003, 0.089]	1.044 [0.997, 1.093]	0.07						

MV-LASSO	depression	0.097 [0.041, 0.153]	1.102 [1.042, 1.165]	0.001						

	panic	0.04 [0.000, 0.081]	1.041 [1.000, 1.084]	0.053						

MV-median	depression	0.079 [0.004, 0.155]	1.082 [1.004, 1.168]	0.04						

	panic	0.05 [–0.014, 0.113]	1.051 [0.986, 1.120]	0.123						

Models with mutual adjustment for anxiety and panic										

MV-IVM	anxiety	0.705 [–0.115, 1.526]	2.024 [0.891, 4.600]	0.092	42	67.5684	0.0041			

	panic	0.087 [0.009, 0.166]	1.091 [1.009, 1.181]	0.029						

MV-Egger	anxiety	0.563 [–0.73, 1.856]	1.756 [0.482, 6.389]	0.394		67.4307	0.0031	0	0	0.778

	panic	0.085 [0.004, 0.166]	1.089 [1.004, 1.181]	0.04						

MV-LASSO	anxiety	0.463 [–0.207, 1.134]	1.589 [0.813, 3.108]	0.175						

	panic	0.113 [0.047, 0.180]	1.120 [1.048, 1.197]	0.001						

MV-median	anxiety	0.349 [–0.609, 1.306]	1.418 [0.544, 3.691]	0.476						

	panic	0.056 [–0.04, 0.152]	1.058 [0.961, 1.164]	0.252						


**Table 3:** MVMR estimating the associations of each mediator with hypertension with adjustment for depression.


METHOD	EXPOSURE	β (95%CI)	OR (95%CI)	SE	P	NO OF SNPs	MVMR HETEROGENEITY TEST (IVW/EGGER)		MVMR DIRECTIONAL PLEIOTROPY TEST		
	
							Q	P	EGGER INTERCEPT	SE	P

T2DM											

MV-IVM	depression	0.122[0.017, 0.227]	1.130[1.017, 1.255]	0.054	0.023	37	116.3391	<0.0001			

	T2DM	0.01[0.005, 0.015]	1.010[1.005, 1.015]	0.003	<0.001						

MV-Egger	depression	0.126[–0.016, 0.269]	1.134[0.984, 1.309]	0.073	0.083		116.3096	<0.0001	0	0	0.926

	T2DM	0.01[0.005, 0.015]	1.010[1.005, 1.015]	0.003	<0.001						

MV-LASSO	depression	0.097[0.010, 0.184]	1.102[1.010, 1.202]	0.044	0.029						

	T2DM	0.011[0.007, 0.016]	1.011[1.007, 1.016]	0.002	<0.001						

MV-median	depression	0.143[0.033, 0.253]	1.154[1.034, 1.288]	0..56	0.011						

	T2DM	0.012[0.007, 0.018]	1.012[1.007, 1.018]	0.003	<0.001						

Smoking											

MV-IVM	depression	0.127[0.043, 0.211]	1.135[1.044, 1.235]	0.043	0.003	23	54.0476	0.0001			

	smoking	0.036[0.015, 0.058]	1.037[1.015, 1.060]	0.011	0.001						

MV-Egger	depression	0.188[0.036, 0.34]	1.207[1.037, 1.405]	0.078	0.015		51.7048	0.0001	0	0	0.341

	smoking	0.04[0.017, 0.063]	1.041[1.017, 1.065]	0.012	0.001						

MV-LASSO	depression	0.148[0.088, 0.208]	1.160[1.092, 1.231]	0.031	<0.001						

	smoking	0.032[0.010, 0.054]	1.033[1.010, 1.055]	0.011	0.004						

MV-median	depression	0.148[0.064, 0.231]	1.160[1.066, 1.260]	0.043	0.001						

	smoking	0.044[0.016, 0.073]	1.045[1.016, 1.076]	0.014	0.002						


### Effect of depression on each mediator

Of seven candidate mediators, two risk factors met the screening criteria and were included in mediation MR analyses (Figure 1). In UVMR analyses, a higher risk of depression was associated with a higher risk of T2DM (IVW OR: 4.730, 95%CI: [1.321, 16.936], p < 0.05), increased smoking rate (IVW β: 0.355, 95%CI: [0.101, 0.610], p < 0.01), and elevated triglycerides levels (IVW β: 0.792, 95%CI: [0.506, 1.007], p < 0.001) (Table S8). However, depression did not exhibit a potential causal relationship with BMI, HDL, blood urea nitrogen levels, and acute renal failure, failing to meet the mediation screening criterion (1) (Table S8). Genetic instrumental variables for depression displayed consistent heterogeneity with TG, while there was no heterogeneity with other mediators (Table S6). All genetic instrumental variables for mediators have no pleiotropy (Table S7).

In the bidirectional MR analysis, a higher triglycerides level was associated with a higher risk of depression (IVW β: 0.008, 95%CI: [0.001, 0.016], p < 0.05), which did not meet mediation screening criterion (1) (Table S9). As shown in Table S10, the association between depression and T2DM or smoking aligns directionally with that between T2DM or smoking and hypertension, satisfying the mediation screening criterion (3). Considering these results, we selected T2DM and smoking as mediators for subsequent analysis.

### Effect of each mediator on hypertension and mediating effects

**Figure 3:**
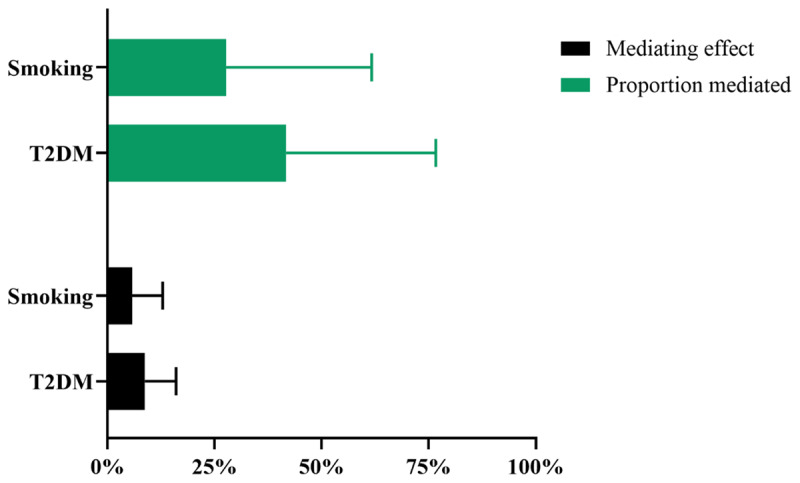
Mendelian randomization estimates of mediated effect and proportions mediated by mediators in the causal association between depression and hypertension. T2DM: type 2 diabetes mellitus.

In the UVMR analysis, higher risk of T2DM (OR: 1.012, 95%CI: [1.008, 1.032], p < 0.01) and smoking (IVW β: 0.036, 95%CI: [0.011, 0.060], p < 0.01) are strongly correlated with a higher risk of hypertension, and these results are stable after sensitivity analysis (Table S10). The genetic instrumental variables for T2DM show persistent heterogeneity with the outcome (Table S6), and both do not exhibit horizontal pleiotropy (Table S7). In the MVMR analysis, the causal relationship between T2DM and hypertension persists after adjusting for depression (OR: 1.010, 95%CI: [1.005, 1.015], p < 0.001), and the causal relationship between smoking and hypertension persists after adjusting for depression (OR: 1.037, 95%CI: [1.015, 1.060], p < 0.001) (Table 3). All directions and statistical significances of the IVW results in MVMR are consistent with the results of the MV-Egger, MV-median, and MV-Lasso regression analysis, and the MVMR Egger intercept analysis shows that there is no pleiotropy in the instrumental variables (Table 3). T2DM and smoking have mediation effects of 8.71% and 5.79% between depression and hypertension, with mediation proportions of 41.7% and 27.7% (Figure 3), respectively.

## Discussion

To the best of our knowledge, this study is the first MR analysis to investigate the potential causal relationship between depression, T2DM, smoking, and hypertension using an MVMR approach. Our findings from the Mendelian analysis indicated a bidirectional causal relationship of genetic liability to depression on hypertension risk, with or without adjusting for anxiety and panic attacks. In addition, this study has demonstrated that T2DM and smoking played a mediating role in this pathway from depression to hypertension.

Our MR results confirmed the positive correlation between depression and hypertension, indicating that depression can increase the incidence of hypertension, and vice versa. This finding aligns well with multiple epidemiological investigations. Numerous studies have reported that those suffering from depression face a heightened risk of developing hypertension (29). For instance, Qiu and colleagues discovered that depressive symptoms were significantly associated with an elevated risk of incident hypertension (30). Moreover, clinical research has consistently shown a higher prevalence of hypertension among depressed patients (31). Similarly, existing literature mostly supports the hypothesis that hypertension can contribute to the onset of depression. A cross-sectional study suggested a significant increase in depressive symptoms among subjects who were aware of their hypertensive condition compared to those who were not (32). Gan et al. also reported a high incidence of depression in elderly hypertensive patients (33). However, the scientific landscape is not without conflict. Some studies have failed to support the notion that hypertension serves as a risk factor for depression (34,35). A prospective cohort study has demonstrated a bidirectional association between depression and hypertension. Their results indicated that while depressive symptoms were associated with an increased risk of hypertension, hypertension was associated with a decreased risk of depression (17). This result does not entirely concur with the findings of our study. This discrepancy may be primarily ascribed to two main factors. Firstly, there are variations in the study populations. The previous study focused on Asian participants, whereas our study centered on individuals of European descent. Secondly, differences in research methodologies play a crucial role. The previous studies were observational in nature, merely reflecting associations. In contrast, our current study employed Mendelian randomization, a more robust approach, to assess the causal relationship between depression and hypertension. This method provides a stronger foundation for inferring causality compared to observational studies. In summary, while there are similarities and differences between our study and previous research, the use of Mendelian randomization in our study offers a unique perspective on the causal relationship between depression and hypertension. Further research is needed to reconcile the conflicting findings and gain a more comprehensive understanding of this complex association.

Our study generally supports previous findings from MR studies while also assessing several novel associations. For instance, Zhang and colleagues reported an association between genetically predicted major depressive disorder and the risk of hypertension in European populations (18), and our study successfully replicated this finding. Another bidirectional two-sample Mendelian randomization indicated that depression acts as a causal risk factor for hypertension, but the reverse relationship may not hold true (19). Intriguingly, our results suggest a bidirectional causal relationship between the two conditions. Conversely, yet another Mendelian randomization investigation failed to detect any association between blood pressure and depressive symptoms (20).

Our findings from Mendelian analysis indicate a significant association between anxiety and an increased risk of subsequent hypertension. This result is in line with numerous studies that have consistently reported an increased susceptibility to hypertension among patients suffering from anxiety or panic (36,37,38). A recent systematic review and meta-analysis further confirmed a significant positive correlation between anxiety and a heightened risk of hypertension (39). This suggests that anxiety may act as a precursor to hypertension, a notion that is bolstered by theories regarding causal mechanisms (39). Shah et al. pointed out that participants with symptoms of depression or anxiety were more likely to develop hypertension (40). Additionally, another study found that anxiety was associated with a 2.18-fold increase in the risk of hypertension (41). However, a cross-sectional study detected no differences in the prevalence of panic, anxiety, and depression between resistant hypertensive patients and non-resistant control groups (16). In contrast, Davies et al. observed that the incidence of panic attacks was significantly higher in hypertensive patients compared to normotensive individuals (42).

Several potential mechanisms may help explain the impact of mental disorder on the occurrence of hypertension. Some studies have indicated that mental disorder may trigger hypertension by stimulating the sympathetic nervous system and inhibiting the parasympathetic nervous system (39,43). Another way in which mental disorder can lead to hypertension is via the hypothalamic-pituitary-adrenal axis (44). Furthermore, depression can elevate the risk of hypertension by promoting unhealthy lifestyles such as smoking and contributing to obesity (45,46).

In our study, we utilized mediation MR analysis to systematically investigate the mediating roles of T2DM and smoking in the relationship between depression and hypertension. Traditional observational studies have reported an alarmingly high prevalence of hypertension among patients with T2DM (47,48). The underlying molecular and cellular mechanisms involve the inappropriate activation of the renin-angiotensin-aldosterone system and the sympathetic nervous system, enhanced activation of renal and endothelial sodium channels, mitochondria dysfunction, oxidative stress, inflammation, abnormal gut microbiota, and increased renal sodium-glucose cotransporter 2 activity (49). Additionally, prior research has demonstrated that depression increases the likelihood of developing T2DM and subsequent insulin resistance (50,51). Our UVMR results suggested that a significant positive association between a higher risk of depression and an increased risk of T2DM, with no evidence of a reverse relationship.

Numerous studies have reported a positive association between smoking and mental illness. However, the direction of this association remains ambiguous in the literature. A systematic review revealed that nearly half of the studies found an association between baseline depression or anxiety and subsequent smoking behavior (52). Our UVMR analyses indicated that a higher risk of depression is significantly associated with an elevated smoking rate, without evidence of the reverse association. Several hypotheses have been proposed to explain the high rates among individuals with depression and anxiety. The self-medication hypothesis postulates that individuals turn to smoking to alleviate their symptoms, suggesting that symptoms of depression and anxiety may drive smoking behavior (52).

This study also has several limitations. Firstly, it was based on summary data from individuals of European ancestry, which restricts the generalizability of our findings to other ethnic groups. Secondly, there was sample overlap between the exposure and outcome GWASs in several analyses, which might have led to potential overfitting and bias in the MR estimates. Thirdly, evidence of weak instrument bias may still exist. Finally, the heterogeneity among SNPs could introduce potential bias and undermine the robustness of our MR results. Given these limitations, future studies are needed to further explore the causal relationship between mental disorders and hypertension.

## Conclusions

To the best of our knowledge, this study is the first Mendelian randomization study to assess the mediating roles of T2DM and smoking on the association between depression and hypertension. By delving into these complex associations, our investigation not only provides a crucial foundation for the future formulation of more effective treatment strategies but also offers valuable perspectives on the underlying pathophysiological mechanisms linking depression and hypertension. Further studies are needed to determine whether preventing depression, regulating type 2 diabetes mellitus, and controlling smoking can reduce the incidence of hypertension.

## Data Accessibility Statement

The datasets analyzed during the current study are available in the IEU open GWAS project at https://gwas.mrcieu.ac.uk/datasets/ and GWAS catalog at https://www.ebi.ac.uk/gwas/.

## Additional File

The additional file for this article can be found as follows:

10.5334/gh.1483.s1 Supplementary Material. Tables S1 to S10 and Figures S1 to S3.

## Figures and Tables

**Table:** 

Abbreviation	Term
95%CI	95% confidence intervals
BMI	Body mass index
BUN	Blood urea nitrogen
HDL	High density lipoprotein
IVs	Instrumental variables
IVW	Inverse variance weighting
GWAS	Genome-wide association studies
MR	Mendelian randomization
MVMR	Multivariable Mendelian randomization
OR	Odds ratios
SNPs	Single nucleotide polymorphisms
T2DM	Type 2 diabetes mellitus
TG	Triglycerides
UVMR	Univariate Mendelian randomization
