# Supplementary material for: Exploring the Causal Relationships and Mediating Factors Between Mental Disorders and Hypertension: A Multivariable Mendelian Randomization Study
Source: Glob Heart. 2025 Oct 14;20(1):94. doi: 10.5334/gh.1483 (PMC12533420; doi:10.5334/gh.1483)
Supplement: Supplementary Material. — Tables S1 to S10 and Figures S1 to S3. [file gh-20-1-1483-s1.pdf]

Supplementary material

Table S1 Detailed information on the SNPs selected for depression

SNP	Effect_allele	Other_allele	Beta	SE	P value	EAF	F
rs10127497	T	A	0.0097175	0.0017084	1.29E-08	0.138233	32.3541484
rs2568958	A	G	0.0086706	0.0011991	4.81E-13	0.599523	52.28625021
rs7548151	A	G	0.012507	0.0021234	3.87E-09	0.0836238	34.69306531
rs30266	A	G	0.007785	0.0012541	5.38E-10	0.328688	38.53478126
rs11961509	G	A	0.014247	0.0025342	1.89E-08	0.0572851	31.6056754
rs3132685	A	G	-0.013055	0.0017833	2.47E-13	0.130202	53.5926131
rs112348907	G	A	0.0073444	0.0012977	1.52E-08	0.295904	32.03052334
rs3807865	A	G	0.0081747	0.001193	7.28E-12	0.411722	46.95293662
rs2402273	C	T	0.0072069	0.0012008	1.95E-09	0.409436	36.02098907
rs263575	A	G	-0.0065899	0.0011794	2.31E-08	0.460081	31.22017994
rs1021363	G	A	-0.0070386	0.0012298	1.04E-08	0.6422	32.75696019
rs10501696	G	A	-0.0078805	0.0012074	6.73E-11	0.498721	42.59956886
rs9530139	T	C	-0.008854	0.0014872	2.63E-09	0.195464	35.44380037
rs28541419	G	C	-0.0078206	0.0014078	2.78E-08	0.23097	30.8601637

Table S2 Detailed information on the SNPs selected for anxiety

SNP	Effect_allele	Other_allele	Beta	SE	P value	EAF	F
rs4844689	T	C	0.000533369	0.000130408	4.30E-05	0.305067	16.72811756
rs10910126	G	C	-0.000544643	0.00012495	1.30E-05	0.349423	18.99990072
rs62142330	G	T	-0.000563649	0.000137487	4.10E-05	0.253677	16.8071552
rs10445754	A	T	-0.000560633	0.000126457	9.30E-06	0.658122	19.65493292
rs1949363	T	C	-0.000490674	0.000121027	5.00E-05	0.585729	16.43696217
rs11132551	C	T	-0.000561252	0.0001201	3.00E-06	0.567631	21.83885116
rs12109689	G	T	-0.000535279	0.000123707	1.50E-05	0.446648	18.72284582
rs4413606	G	A	-0.000513366	0.000124206	3.60E-05	0.630128	17.08319321
rs952884	C	T	0.000504939	0.000123676	4.50E-05	0.368037	16.66890111
rs606368	T	G	0.000495601	0.000122135	5.00E-05	0.606019	16.46584696
rs2928627	C	T	0.000549562	0.000120769	5.40E-06	0.571097	20.70725112
rs7097102	C	T	0.000578662	0.000122889	2.50E-06	0.385066	22.17297192
rs2166153	C	T	-0.000504647	0.000121805	3.40E-05	0.397366	17.16505342
rs4901598	T	C	0.00049223	0.000119136	3.60E-05	0.518889	17.0706528
rs11159379	T	C	0.000521212	0.000123979	2.60E-05	0.472108	17.67390653
rs7225123	C	T	-0.00051682	0.000122987	2.60E-05	0.497395	17.65876014
rs12458570	A	G	0.000593744	0.000130263	5.20E-06	0.703586	20.77573112
rs7410377	A	G	-0.000687331	0.000141296	1.10E-06	0.238169	23.66312669

Table S3 Detailed information on the SNPs selected for panic

SNP	Effect_allele	Other_allele	Beta	SE	P value	EAF	F
rs10244964	T	G	-0.0109008	0.00247248	1.04E-05	0.0377928	19.43798169
rs1033807	A	G	0.00521708	0.00124287	2.70E-05	0.173653	17.6199058
rs10468145	A	C	-0.00398678	0.000957861	3.15E-05	0.407376	17.32365634
rs10510943	A	G	-0.0080107	0.00188944	2.24E-05	0.0684275	17.97524113
rs1072841	C	A	0.00467074	0.00105041	8.73E-06	0.281053	19.7721406
rs10890292	C	G	0.00478535	0.0010647	6.98E-06	0.265582	20.20100178
rs11120008	T	C	0.0085359	0.00210418	4.98E-05	0.0561726	16.45632518
rs113351744	A	G	-0.0153484	0.00375696	4.40E-05	0.0171638	16.68987468
rs113675006	G	A	-0.010964	0.00266274	3.83E-05	0.0349965	16.95432598
rs114217210	T	G	0.0100757	0.00228756	1.06E-05	0.0441269	19.40016699
rs114407185	C	T	0.0255254	0.00591277	1.58E-05	0.00685257	18.63644798
rs115637887	C	T	0.025203	0.00566684	8.70E-06	0.00809326	19.77983103
rs115962393	G	A	0.00982455	0.00214152	4.49E-06	0.0520215	21.04655725
rs11664902	A	G	0.0125688	0.00293494	1.85E-05	0.0266172	18.33957118
rs117376553	T	C	0.0142786	0.00329369	1.46E-05	0.0239808	18.79341969
rs11812217	A	T	-0.00949839	0.0021818	1.34E-05	0.049334	18.95265776
rs12120173	G	T	-0.00441053	0.0010628	3.33E-05	0.279781	17.22179715
rs12358669	C	T	0.0105825	0.00221524	1.78E-06	0.0581313	22.82101649
rs12385751	T	C	0.00613021	0.00134803	5.43E-06	0.14396	20.68005025
rs12485997	A	G	0.00443133	0.00106178	3.00E-05	0.27518	17.41803283
rs12592125	A	G	-0.00392841	0.000952485	3.72E-05	0.420949	17.01050965
rs12610082	A	C	-0.00482189	0.00118547	4.76E-05	0.803357	16.54449295
rs12750925	C	T	0.00412837	0.000964737	1.88E-05	0.597764	18.31215083
rs191413120	G	T	0.0207883	0.00488605	2.10E-05	0.010314	18.10181564

Continued

SNP	Effect_allele	Other_allele	Beta	SE	P value	EAF	F
rs12935880	A	G	0.0106834	0.00242518	1.06E-05	0.0390762	19.40577637
rs12970393	G	A	0.00420402	0.00098392	1.93E-05	0.643472	18.25618256
rs140163380	G	C	-0.00880726	0.00187085	2.51E-06	0.0686317	22.16173198
rs140338545	T	C	0.0219909	0.00491632	7.72E-06	0.00982225	20.0080941
rs140457556	T	C	0.015198	0.0036387	2.96E-05	0.0181145	17.44537779
rs142872233	T	C	-0.0137543	0.00327024	2.60E-05	0.0210694	17.6895886
rs143068958	G	C	0.0184126	0.00398027	3.73E-06	0.0144208	21.3995761
rs143869074	G	T	-0.0177918	0.00424234	2.74E-05	0.0125073	17.58850118
rs144480991	C	T	0.0220181	0.00540341	4.61E-05	0.00816283	16.60442479
rs1451125	A	T	0.00436468	0.00102279	1.98E-05	0.302618	18.21091934
rs145980608	A	G	0.0110749	0.00273056	5.00E-05	0.0312263	16.45038926
rs146405975	C	T	0.012435	0.00291495	1.99E-05	0.0300147	18.19823881
rs146686945	G	A	0.025096	0.00559506	7.29E-06	0.00848147	20.11868149
rs147504893	A	G	0.0187876	0.00458279	4.14E-05	0.0113896	16.80670963
rs148693942	G	C	0.0166138	0.00404681	4.04E-05	0.0140788	16.85436237
rs150941544	A	G	0.0279275	0.00543709	2.80E-07	0.00856884	26.38341915
rs1543163	C	A	0.00672656	0.00165419	4.78E-05	0.911103	16.53542107
rs1607566	C	T	-0.00404028	0.000956847	2.42E-05	0.529472	17.82944926
rs16875314	C	G	0.00821582	0.00197624	3.22E-05	0.060427	17.28313255
rs16968353	A	T	0.00957688	0.00226937	2.44E-05	0.0457249	17.80891547
rs17233820	G	C	0.00701189	0.00165581	2.29E-05	0.0894185	17.93284466
rs182627027	A	G	0.0176604	0.00426339	3.44E-05	0.013039	17.15895943
rs183132083	T	C	-0.00919939	0.00224481	4.17E-05	0.0474653	16.79418314
rs1884497	A	G	-0.00425201	0.00104482	4.71E-05	0.285803	16.56172617

Continued

SNP	Effect_allele	Other_allele	Beta	SE	P value	EAF	F
rs191707533	T	C	0.0275257	0.00609173	6.23E-06	0.00673892	20.41716564
rs192582121	T	A	0.0235951	0.00545144	1.50E-05	0.00827191	18.73359692
rs2045087	A	G	0.00402038	0.000940738	1.92E-05	0.511949	18.26403931
rs208684	A	C	-0.00454047	0.00111409	4.59E-05	0.760593	16.60967248
rs2128632	A	G	0.00428832	0.000952573	6.74E-06	0.515951	20.2664572
rs35171419	T	C	0.0109474	0.00238811	4.56E-06	0.0403845	21.01422216
rs35532855	T	C	-0.0121094	0.00295649	4.21E-05	0.026392	16.77615467
rs3791357	T	C	0.00616922	0.00147712	2.96E-05	0.117785	17.4433124
rs4662732	C	T	-0.00581103	0.00104979	3.11E-08	0.280772	30.64088974
rs4760644	G	A	0.00439152	0.00102447	1.82E-05	0.67509	18.37516465
rs4896494	A	G	-0.00415054	0.000989413	2.73E-05	0.650309	17.59762192
rs520836	C	T	0.00434911	0.00096042	5.95E-06	0.400601	20.50587916
rs55638979	A	G	0.00755542	0.00178646	2.35E-05	0.0798378	17.88671676
rs58292930	A	G	0.00517841	0.00106696	1.22E-06	0.262536	23.55573116
rs58597889	T	C	-0.00841349	0.00194803	1.57E-05	0.0630521	18.65353233
rs58659940	C	T	-0.00601061	0.00144276	3.10E-05	0.120293	17.35597088
rs60313149	G	C	-0.00415423	0.000976345	2.09E-05	0.379496	18.10399672
rs6061519	C	T	-0.00385464	0.000949091	4.88E-05	0.474106	16.49498547
rs61322610	C	T	0.0153768	0.00339108	5.78E-06	0.0196952	20.56155049
rs61743199	G	A	-0.00793282	0.00181449	1.23E-05	0.0720057	19.11375619
rs61807674	G	A	0.00768985	0.001564	8.81E-07	0.100544	24.1747638
rs62041744	A	G	0.00832151	0.00201926	3.77E-05	0.0637268	16.98321056
rs62315074	T	C	-0.0145933	0.00340341	1.81E-05	0.0211111	18.38562847
rs62432286	G	C	-0.00927395	0.00207778	8.07E-06	0.0554727	19.92188263

Continued

SNP	Effect_allele	Other_allele	Beta	SE	P value	EAF	F
rs6428569	G	A	-0.00406016	0.000942511	1.65E-05	0.494413	18.55724244
rs6563519	T	C	-0.00773728	0.00178209	1.42E-05	0.924642	18.85026069
rs7027184	C	G	-0.010237	0.00235744	1.41E-05	0.0416539	18.85663133
rs7094449	G	A	0.00809698	0.00192298	2.55E-05	0.065567	17.72950451
rs71612841	T	G	0.0136706	0.00296639	4.06E-06	0.0263509	21.23824641
rs72550256	A	G	0.00527808	0.00126139	2.86E-05	0.172002	17.5086705
rs72670500	A	G	0.0237585	0.00580592	4.28E-05	0.00784729	16.745416
rs72854507	T	C	0.021904	0.00469017	3.01E-06	0.012637	21.81070444
rs74494412	A	C	0.0171314	0.00406694	2.53E-05	0.0138774	17.74394491
rs74791474	A	G	0.0122596	0.00290084	2.38E-05	0.0268763	17.86097038
rs7563956	A	G	-0.00408194	0.000963441	2.27E-05	0.593662	17.95076599
rs7626282	C	A	-0.00665408	0.00148536	7.48E-06	0.113609	20.06839253
rs76771399	T	C	-0.0223759	0.00530949	2.51E-05	0.0083117	17.7605091
rs77567348	A	G	0.015751	0.00357774	1.07E-05	0.0184639	19.38200539
rs78438550	A	G	0.0105971	0.00237206	7.92E-06	0.0415533	19.95826225
rs79045984	A	G	0.0251236	0.00615576	4.48E-05	0.00675593	16.65713793
rs7933552	G	T	-0.00446901	0.000974966	4.57E-06	0.414689	21.01085431
rs80083181	G	T	-0.00804436	0.00194917	3.68E-05	0.0623347	17.03270242
rs80341196	A	G	-0.0148454	0.00332999	8.28E-06	0.0209014	19.87457952
rs828922	C	T	-0.00406209	0.00100138	4.98E-05	0.327961	16.45512768
rs899565	C	A	-0.00412805	0.00101429	4.71E-05	0.406712	16.56401482
rs9540740	A	G	0.00625115	0.00149474	2.89E-05	0.119211	17.48994836
rs971168	T	C	-0.00575367	0.00132139	1.34E-05	0.850009	18.9595431
rs9865108	C	T	0.00674635	0.0014729	4.65E-06	0.116112	20.9793106

Table S4 UVMR assessing the causal association between exposures and hypertension

Exposure	Method	No of SNPs	β (95%CI)	OR (95%CI)	P value
Depression	MR Egger	14	0.045 [-0.248, 0.338]	1.046 [0.780, 1.402]	7.69E-01
	Weighted median	14	0.129 [0.058, 0.199]	1.137 [1.057, 1.223]	3.25E-04
	Inverse variance weighted	14	0.131 [0.072, 0.191]	1.140 [1.075, 1.210]	1.31E-05
	Simple mode	14	0.120 [-0.009, 0.248]	1.127 [0.987, 1.287]	9.11E-02
	Weighted mode	14	0.107 [-0.031, 0.244]	1.113 [0.969, 1.278]	1.51E-01
Anxiety	MR Egger	18	0.060 [-8.917, 9.037]	1.062 [0.000, 8412.101]	9.90E-01
	Weighted median	18	0.533 [-0.350, 1.417]	1.705 [0.704, 4.125]	2.37E-01
	Inverse variance weighted	18	0.986 [0.283, 1.688]	2.679 [1.328, 5.408]	5.94E-03
	Simple mode	18	1.680 [0.084, 3.276]	5.363 [1.087, 26.458]	5.48E-02
	Weighted mode	18	1.680 [-0.045, 3.404]	5.363 [0.956, 30.086]	7.33E-02
Panic	MR Egger	96	-0.003[-0.078, 0.072]	0.997[0.925, 1.075]	9.36E-01
	Weighted median	96	0.042[-0.008, 0.092]	1.043[0.992, 1.096]	9.68E-02
	Inverse variance weighted	96	0.052[0.016, 0.088]	1.054[1.016, 1.092]	4.75E-03
	Simple mode	96	0.036[-0.092, 0.164]	1.037[0.912, 1.178]	5.83E-01
	Weighted mode	96	0.034[-0.091, 0.158]	1.034[0.913, 1.171]	5.98E-01

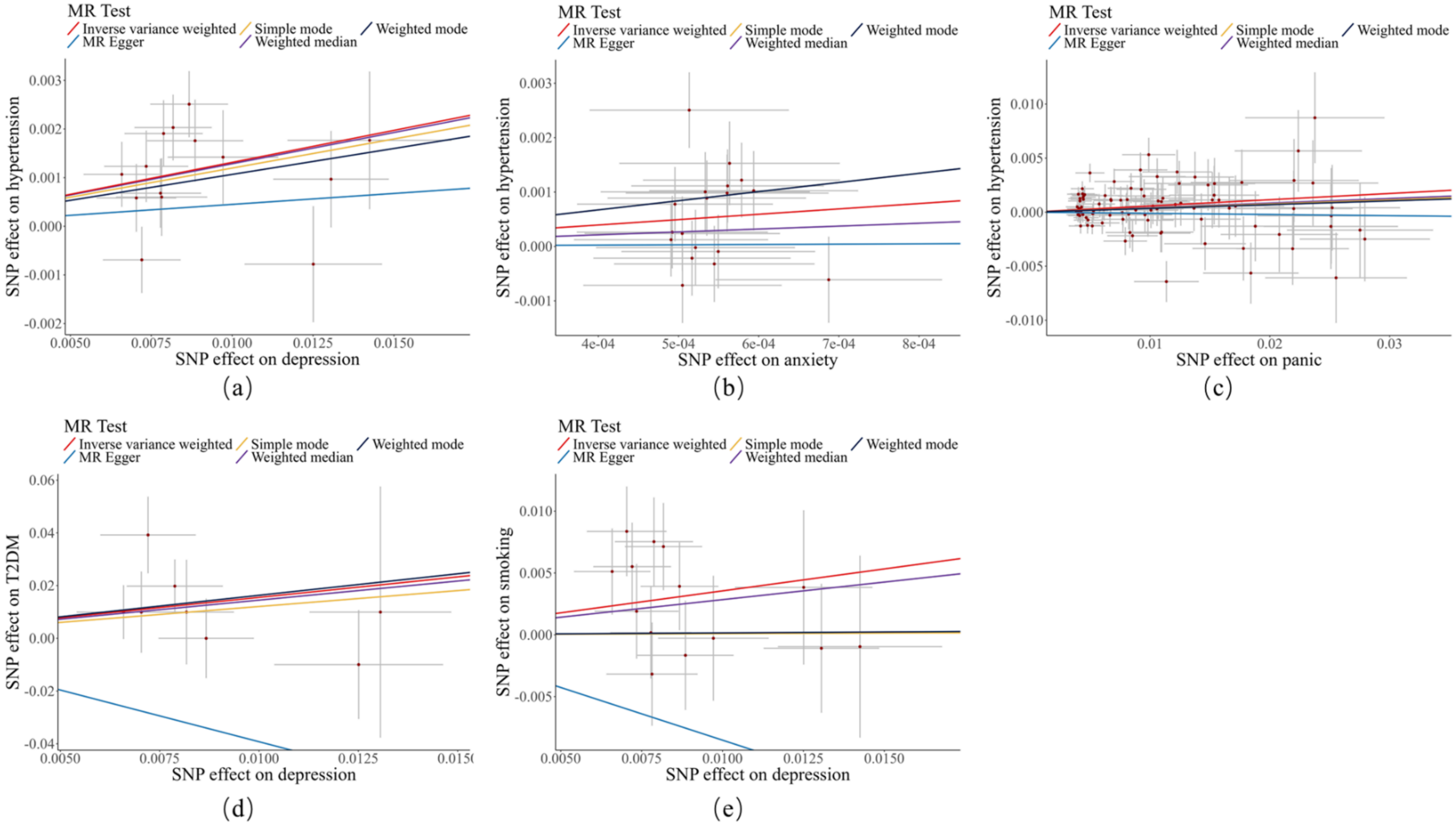

Fig. S1 Scatter plots. The slope of different colorful lines represent the estimated MR effect of different MR methods. (a) Depression to hypertension. (b) Anxiety to hypertension. (c) Panic to hypertension. (d) Depression to T2DM. (e) Depression to smoking.

Table S5 UVMR assessing the causal association between hypertension and exposures, mediators

Outcome	Method	No of SNPs	β (95%CI)	OR (95%CI)	P value
Depression	MR Egger	67	-0.254 [-0.565, 0.057]	0.776 [0.568, 1.059]	1.15E-01
	Weighted median	67	0.026 [-0.068, 0.12]	1.026 [0.934, 1.128]	5.89E-01
	Inverse variance weighted	67	0.096 [0.009, 0.184]	1.101 [1.009, 1.202]	3.10E-02
	Simple mode	67	-0.01 [-0.214, 0.194]	0.99 [0.807, 1.214]	9.21E-01
	Weighted mode	67	0.001 [-0.165, 0.168]	1.001 [0.848, 1.183]	9.88E-01
Anxiety	MR Egger	46	0.009 [-0.024, 0.042]	1.009 [0.976, 1.043]	6.00E-01
	Weighted median	46	0.009 [-0.001, 0.019]	1.009 [0.999, 1.019]	8.98E-02
	Inverse variance weighted	46	0.006 [-0.001, 0.014]	1.006 [0.999, 1.014]	9.07E-02
	Simple mode	46	0.015 [-0.006, 0.036]	1.015 [0.994, 1.036]	1.75E-01
	Weighted mode	46	0.011 [-0.008, 0.029]	1.011 [0.992, 1.03]	2.57E-01
Panic	MR Egger	69	-0.094 [-0.288, 0.1]	0.91 [0.749, 1.105]	3.44E-01
	Weighted median	69	0.01 [-0.063, 0.084]	1.01 [0.939, 1.087]	7.86E-01
	Inverse variance weighted	69	0.017 [-0.035, 0.07]	1.018 [0.966, 1.072]	5.12E-01
	Simple mode	69	-0.074 [-0.268, 0.12]	0.929 [0.765, 1.127]	4.57E-01
	Weighted mode	69	-0.093 [-0.252, 0.066]	0.911 [0.777, 1.068]	2.55E-01
T2DM	MR Egger	23	-5.338 [-12.176, 1.5]	0.005 [0, 4.484]	1.41E-01
	Weighted median	23	1.735 [-0.298, 3.767]	5.667 [0.743, 43.248]	9.43E-02
	Inverse variance weighted	23	0.998 [-0.78, 2.777]	2.714 [0.459, 16.067]	2.71E-01
	Simple mode	23	2.462 [-0.952, 5.877]	11.729 [0.386, 356.566]	1.72E-01
	Weighted mode	23	2.185 [-0.783, 5.152]	8.887 [0.457, 172.717]	1.63E-01

Continued

Outcome	Method	No of SNPs	β (95%CI)	OR (95%CI)	P value
Smoking	MR Egger	3	-1.98 [-5.467, 1.506]	0.138 [0.004, 4.508]	4.66E-01
	Weighted median	3	-0.198 [-0.838, 0.442]	0.821 [0.433, 1.556]	5.45E-01
	Inverse variance weighted	3	-0.182 [-0.712, 0.347]	0.833 [0.491, 1.415]	5.00E-01
	Simple mode	3	-0.257 [-1.008, 0.494]	0.773 [0.365, 1.639]	5.72E-01
	Weighted mode	3	-0.224 [-0.995, 0.546]	0.799 [0.37, 1.726]	6.25E-01

Table S6 Heterogeneity test of the associations of UVMR

Exposure	Outcome	Method	Q stastic	Q df	P value
Depression	Hypertension	MR Egger	19.151	12	8.49E-02
		IVW	19.708	13	1.03E-01
Anxiety	Hypertension	MR Egger	23.412	16	1.03E-01
		IVW	23.472	17	1.34E-01
Panic	Hypertension	MR Egger	112.609	94	9.27E-02
		IVW	115.803	95	7.23E-02
T2DM	Hypertension	MR Egger	41.760	21	4.51E-03
		IVW	41.773	22	6.66E-03
Smoking	Hypertension	MR Egger	8.157	5	1.48E-01
		IVW	8.751	6	1.88E-01
Depression	T2DM	MR Egger	4.510	6	6.08E-01
		IVW	7.190	7	4.09E-01
Depression	Smoking	MR Egger	9.400	12	6.68E-01
		IVW	13.378	13	4.19E-01
Depression	BMI	MR Egger	10.107	6	1.20E-01
		IVW	13.291	7	6.53E-02
Depression	TG	MR Egger	18.872	7	8.60E-03
		IVW	20.094	8	9.99E-03
Depression	HDL	MR Egger	14.506	9	1.05E-01
		IVW	17.451	10	6.50E-02
Depression	BUN	MR Egger	7.943	7	3.38E-01
		IVW	9.217	8	3.24E-01

Continued

Exposure	Outcome	Method	Q stastic	Q df	P value
Depression	Renal failure	MR Egger	6.666	6	3.53E-01
		IVW	6.884	7	4.41E-01
Hypertension	Depression	MR Egger	124.644	65	1.24E-05
		IVW	134.721	66	1.27E-06
Hypertension	Anxiety	MR Egger	37.422	44	7.48E-01
		IVW	37.447	45	7.81E-01
Hypertension	Panic	MR Egger	78.408	67	1.61E-01
		IVW	80.019	68	1.51E-01
Hypertension	T2DM	MR Egger	35.784	21	2.31E-02
		IVW	41.767	22	6.67E-03
Hypertension	Smoking	MR Egger	0.372	1	5.42E-01
		IVW	1.418	2	4.92E-01
T2DM	Depression	MR Egger	16.664	20	6.75E-01
		IVW	16.711	21	7.28E-01
Smoking	Depression	MR Egger	14.355	8	7.30E-02
		IVW	16.855	9	5.10E-02
BMI	Depression	MR Egger	71.328	33	1.23E-04
		IVW	71.620	34	1.71E-04
TG	Depression	MR Egger	400.544	272	6.11E-07
		IVW	401.241	273	6.66E-07
HDL	Depression	MR Egger	52.639	42	1.26E-01
		IVW	54.246	43	1.17E-01

Continued

Exposure	Outcome	Method	Q stastic	Q df	P value
BUN	Depression	MR Egger	11.168	8	1.92E-01
		IVW	11.367	9	2.51E-01
Renal failure	Depression	MR Egger	20.267	21	5.04E-01
		IVW	24.600	22	3.17E-01

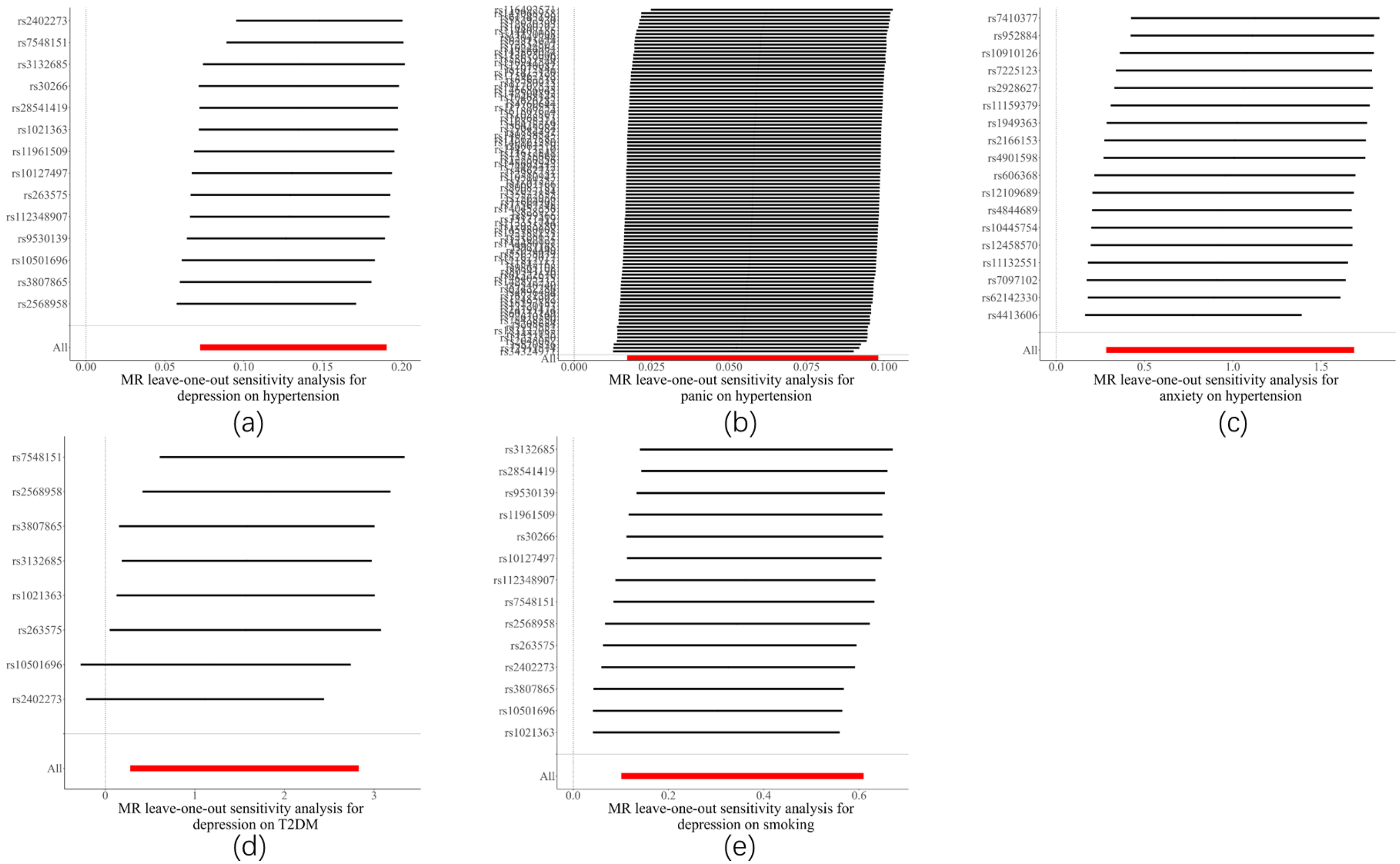

Fig S2. The leave-one-out analysis of MR results of exposures on outcomes. The leave-one-out analysis removes a single SNP each time and calculates the meta-analysis effect of the remaining SNPs to observe whether the results change after removing each SNP, and if a SNP is removed, the results change greatly, indicating that the presence of the SNP has a great impact on the results. In this study, after removing a single SNP each

time, the overall bars did not change much, indicating that the results were reliable and not affected by heterogeneity. T2DM: type 2 diabetes mellitus.

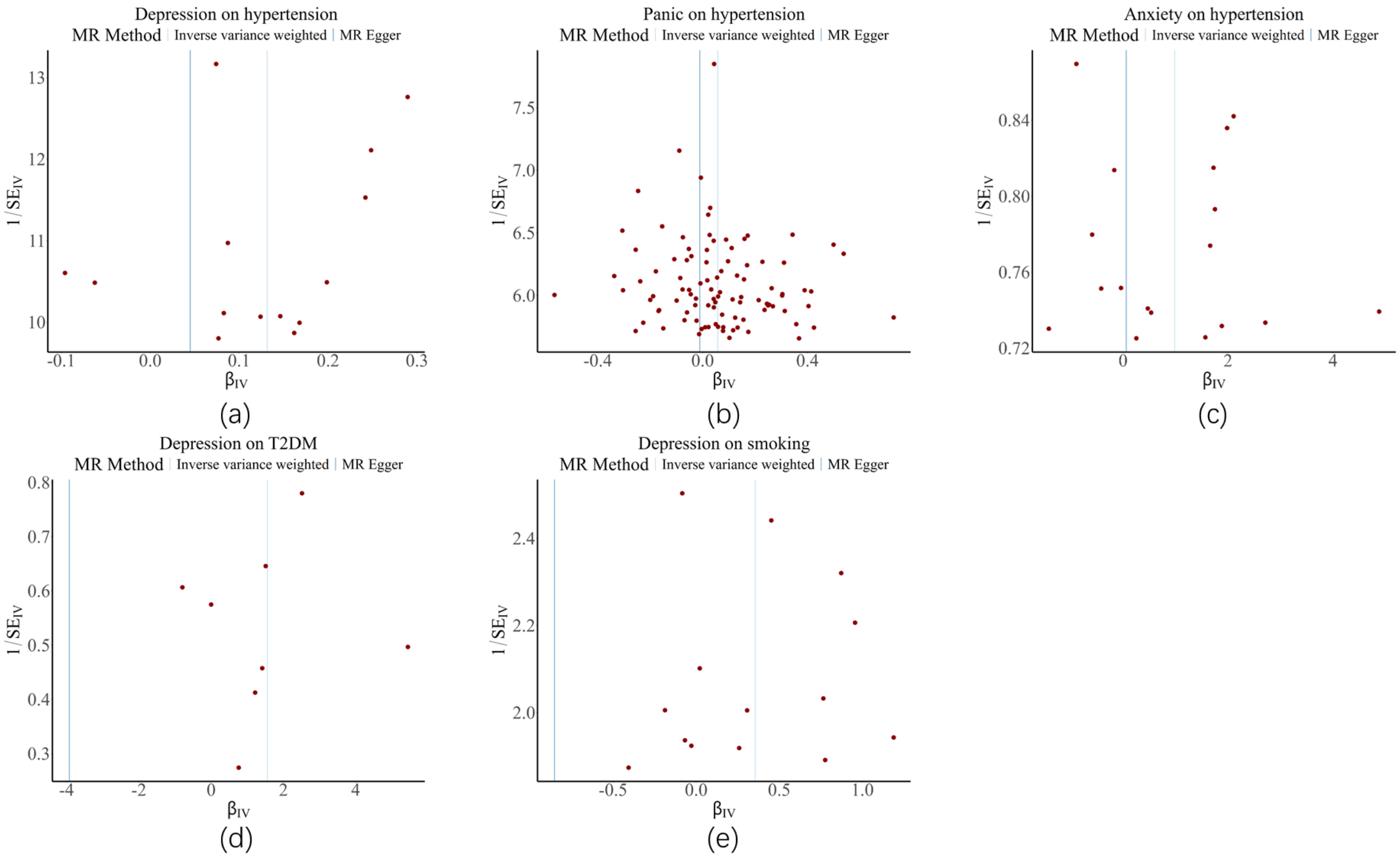

Fig S3. Funnel plots of causal associations. The funnel plots show the Inverse variance weighted and MR Egger MR estimate of each exposure single-nucleotide polymorphism with outcomes versus 1/standard error ($1/SE_{IV}$). (a) depression on hypertension; (b) panic on hypertension; (c) anxiety on hypertension; (d) depression on T2DM; (e) depression on smoking. T2DM: type 2 diabetes mellitus.

Table S7 Directional pleiotropy test of the associations of UVMR

Exposure	Outcome	MR Egger Pleotropy test			MR-PRESSO global pleiotropy test		
		Intercept	SE	P value	RSSobs	P value	Outliers
Depression	Hypertension	7.51E-04	1.27E-03	5.65E-01	23.16121	0.12	None
Anxiety	Hypertension	5.02E-04	2.48E-03	8.42E-01	26.28777	0.144	None
Panic	Hypertension	4.19E-04	2.57E-04	1.06E-01	158.7206	<0.001	rs116492571, rs12911514, rs34324971
T2DM	Hypertension	-6.76E-05	8.44E-04	9.37E-01	107.0713	<0.001	rs1558902, rs5015480, rs6444187
Smoking	Hypertension	8.66E-04	1.44E-03	5.73E-01	52.67024	0.003	rs4900590, rs8042849, rs9358909
Depression	T2DM	4.45E-02	2.72E-02	1.53E-01	9.5911	0.447	None
Depression	Smoking	1.05E-02	5.26E-03	6.93E-02	15.51197	0.454	None
Depression	BMI	6.08E-02	4.42E-02	2.18E-01	48.201	<0.001	rs2568958, rs9530139
Depression	TG	4.29E-03	6.38E-03	5.22E-01	77.32692	<0.001	rs2402273, rs28541419, rs3132685
Depression	HDL	1.38E-02	1.02E-02	2.09E-01	59.90534	<0.001	rs2568958, rs3132685, rs3807865
Depression	BUN	-9.61E-03	9.08E-03	3.25E-01	26.14102	0.02	rs3132685
Depression	Renal failure	2.35E-04	5.29E-04	6.73E-01	8.828233	0.441	None
Hypertension	Depression	1.96E-03	8.54E-04	2.51E-02	146.7845	<0.001	None
Hypertension	Anxiety	-1.35E-05	8.43E-05	8.73E-01	42.567	0.779	None
Hypertension	Panic	6.24E-04	5.32E-04	2.45E-01	85.93473	0.135	None
Hypertension	T2DM	3.41E-02	1.82E-02	7.49E-02	63.23298	0.001	None

Continued

Exposure	Outcome	MR Egger Pleotropy test			MR-PRESSO global pleiotropy test		
		Intercept	SE	P value	RSSobs	P value	Outliers
Hypertension	Smoking	1.56E-02	1.52E-02	4.93E-01	-	-	-
T2DM	Depression	2.50E-04	1.15E-03	8.30E-01	52.40587	0.026	rs571312
Smoking	Depression	1.60E-03	1.36E-03	2.72E-01	20.35291	0.091	None
BMI	Depression	3.38E-04	9.20E-04	7.15E-01	96.54846	<0.001	None
TG	Depression	-1.18E-04	1.71E-04	4.92E-01	489.4093	<0.001	rs2092203, rs213494, rs28752924, rs325485, rs394872
HDL	Depression	5.62E-04	4.96E-04	2.64E-01	55.94198	0.117	None
BUN	Depression	-7.79E-04	2.07E-03	7.16E-01	32.56768	0.005	rs3957147
Renal failure	Depression	-7.44E-03	3.57E-03	4.98E-02	27.09082	0.344	None

Table S8 UVMR assessing the causal association between depression and mediators

Mediators	Method	No of SNPs	β (95%CI)	OR (95%CI)	P value
T2DM	MR Egger	8	-3.923 [-10.599, 2.753]	0.020 [0.000, 15.692]	2.93E-01
	Weighted median	8	1.449 [-0.300, 3.199]	4.261 [0.741, 24.497]	1.04E-01
	Inverse variance weighted	8	1.554 [0.278, 2.829]	4.730 [1.321, 16.936]	1.70E-02
	Simple mode	8	1.207 [-1.480, 3.894]	3.345 [0.228, 49.124]	4.08E-01
	Weighted mode	8	1.632 [-0.736, 4.001]	5.117 [0.479, 54.653]	2.19E-01
Smoking	MR Egger	14	-0.852 [-2.065, 0.361]	0.426 [0.127, 1.434]	1.94E-01
	Weighted median	14	0.284 [-0.069, 0.637]	1.329 [0.934, 1.891]	1.14E-01
	Inverse variance weighted	14	0.355 [0.101, 0.610]	1.426 [1.106, 1.840]	6.22E-03
	Simple mode	14	0.009 [-0.726, 0.744]	1.009 [0.484, 2.103]	9.81E-01
	Weighted mode	14	0.015 [-0.644, 0.675]	1.015 [0.525, 1.963]	9.65E-01
BMI	MR Egger	8	-6.017 [-16.435, 4.401]	0.002 [0.000, 81.555]	3.01E-01
	Weighted median	8	1.920 [-0.930, 4.771]	6.822 [0.394, 117.994]	1.87E-01
	Inverse variance weighted	8	1.082 [-1.54, 3.704]	2.952 [0.214, 40.629]	4.18E-01
	Simple mode	8	2.606 [-3.166, 8.378]	13.542 [0.042, 4349.169]	4.06E-01
	Weighted mode	8	3.193 [-2.645, 9.031]	24.350 [0.071, 8354.635]	3.19E-01
TG	MR Egger	9	0.301 [-1.158, 1.76]	1.351 [0.314, 5.813]	6.98E-01
	Weighted median	9	0.838 [0.563, 1.112]	2.311 [1.757, 3.041]	2.15E-09
	Inverse variance weighted	9	0.792 [0.506, 1.077]	2.207 [1.659, 2.937]	5.59E-08
	Simple mode	9	0.948 [0.497, 1.399]	2.581 [1.644, 4.052]	3.35E-03
	Weighted mode	9	0.926 [0.444, 1.408]	2.525 [1.56, 4.087]	5.48E-03
BUN	MR Egger	9	0.752 [-1.428, 2.933]	2.122 [0.24, 18.776]	5.20E-01
	Weighted median	9	-0.551 [-1.149, 0.047]	0.577 [0.317, 1.048]	7.11E-02
	Inverse variance weighted	9	-0.401 [-0.852, 0.051]	0.67 [0.426, 1.052]	8.22E-02
	Simple mode	9	-0.668 [-1.691, 0.355]	0.513 [0.184, 1.426]	2.36E-01

Continued

Mediators	Method	No of SNPs	β (95%CI)	OR (95%CI)	P value
	Weighted mode	9	-0.645 [-1.534, 0.245]	0.525 [0.216, 1.278]	1.93E-01
HDL	MR Egger	11	-1.427 [-3.869, 1.016]	0.24 [0.021, 2.762]	2.82E-01
	Weighted median	11	0.254 [-0.318, 0.825]	1.289 [0.727, 2.283]	3.85E-01
	Inverse variance weighted	11	0.224 [-0.278, 0.727]	1.252 [0.757, 2.069]	3.82E-01
	Simple mode	11	0.498 [-0.455, 1.452]	1.646 [0.634, 4.272]	3.30E-01
	Weighted mode	11	0.52 [-0.418, 1.459]	1.683 [0.658, 4.301]	3.03E-01
Renal failure	MR Egger	8	-0.029 [-0.165, 0.107]	0.971 [0.848, 1.113]	6.92E-01
	Weighted median	8	0.000 [-0.014, 0.015]	1.000 [0.986, 1.015]	9.50E-01
	Inverse variance weighted	8	0.002 [-0.009, 0.013]	1.002 [0.991, 1.013]	7.50E-01
	Simple mode	8	-0.001 [-0.024, 0.022]	0.999 [0.976, 1.022]	9.17E-01
	Weighted mode	8	-0.001 [-0.021, 0.020]	0.999 [0.979, 1.020]	9.58E-01

Table S9 UVMR assessing the causal association between mediators and depression

Mediators	Method	No of SNPs	β (95%CI)	OR (95%CI)	P value
T2DM	MR Egger	22	-0.004 [-0.026, 0.018]	0.996 [0.974, 1.018]	7.08E-01
	Weighted median	22	-0.003 [-0.011, 0.004]	0.997 [0.989, 1.004]	3.73E-01
	Inverse variance weighted	22	-0.002 [-0.007, 0.004]	0.998 [0.993, 1.004]	4.94E-01
	Simple mode	22	-0.003 [-0.017, 0.011]	0.997 [0.983, 1.011]	6.45E-01
	Weighted mode	22	-0.004 [-0.014, 0.006]	0.996 [0.986, 1.006]	4.59E-01
Smoking	MR Egger	10	-0.019 [-0.094, 0.057]	0.981 [0.910, 1.058]	6.39E-01
	Weighted median	10	0.022 [-0.008, 0.053]	1.023 [0.992, 1.055]	1.53E-01
	Inverse variance weighted	10	0.022 [-0.010, 0.055]	1.023 [0.990, 1.057]	1.79E-01
	Simple mode	10	0.075 [-0.009, 0.158]	1.078 [0.991, 1.172]	1.13E-01
	Weighted mode	10	0.016 [-0.020, 0.051]	1.016 [0.981, 1.052]	4.06E-01
BMI	MR Egger	35	0.000 [-0.011, 0.010]	1.000 [0.989, 1.010]	9.71E-01
	Weighted median	35	0.002 [-0.002, 0.006]	1.002 [0.998, 1.006]	3.72E-01
	Inverse variance weighted	35	0.002 [-0.002, 0.006]	1.002 [0.998, 1.006]	4.07E-01
	Simple mode	35	0.001 [-0.008, 0.010]	1.001 [0.992, 1.010]	8.64E-01
	Weighted mode	35	0.004 [-0.002, 0.009]	1.004 [0.998, 1.009]	1.87E-01
TG	MR Egger	274	0.012 [0.000, 0.023]	1.012 [1.000, 1.024]	5.51E-02
	Weighted median	274	0.011 [0.001, 0.022]	1.012 [1.001, 1.022]	3.41E-02
	Inverse variance weighted	274	0.008 [0.001, 0.016]	1.008 [1.001, 1.016]	2.44E-02
	Simple mode	274	0.002 [-0.021, 0.024]	1.002 [0.979, 1.025]	8.86E-01
	Weighted mode	274	0.009 [-0.001, 0.020]	1.009 [0.999, 1.020]	7.46E-02
BUN	MR Egger	10	0.019 [-0.098, 0.137]	1.020 [0.906, 1.147]	7.54E-01
	Weighted median	10	-0.017 [-0.05, 0.016]	0.983 [0.951, 1.016]	3.14E-01
	Inverse variance weighted	10	-0.002 [-0.03, 0.025]	0.998 [0.970, 1.025]	8.61E-01
	Simple mode	10	-0.025 [-0.084, 0.035]	0.976 [0.920, 1.035]	4.37E-01

Continued

Mediators	Method	No of SNPs	β (95%CI)	OR (95%CI)	P value
	Weighted mode	10	-0.023 [-0.084, 0.038]	0.977 [0.919, 1.038]	4.71E-01
HDL	MR Egger	44	-0.007 [-0.023, 0.009]	0.993 [0.977, 1.009]	3.76E-01
	Weighted median	44	-0.001 [-0.013, 0.011]	0.999 [0.987, 1.011]	9.08E-01
	Inverse variance weighted	44	0.001 [-0.008, 0.009]	1.001 [0.992, 1.009]	8.92E-01
	Simple mode	44	-0.004 [-0.023, 0.015]	0.996 [0.978, 1.015]	7.09E-01
	Weighted mode	44	-0.002 [-0.015, 0.010]	0.998 [0.985, 1.010]	7.04E-01
Renal failure	MR Egger	23	14.738 [1.343, 28.134]	2.52E06 [3.830, 1.65E12]	4.28E-02
	Weighted median	23	0.620 [-0.747, 1.988]	1.860 [0.474, 7.301]	3.74E-01
	Inverse variance weighted	23	0.549 [-0.476, 1.574]	1.732 [0.621, 4.826]	2.94E-01
	Simple mode	23	-1.516 [-4.193, 1.161]	0.220 [0.015, 3.194]	2.79E-01
	Weighted mode	23	1.579 [-1.008, 4.165]	4.848 [0.365, 64.403]	2.44E-01

Table S10 UVMR assessing the causal association between mediators and hypertension

Exposure	Method	No of SNPs	β (95%CI)	OR (95%CI)	P value
T2DM	MR Egger	23	0.013 [-0.004, 0.029]	1.013 [0.996, 1.029]	1.47E-01
	Weighted median	23	0.011 [0.006, 0.016]	1.011 [1.006, 1.017]	1.01E-05
	Inverse variance weighted	23	0.012 [0.008, 0.016]	1.012 [1.008, 1.016]	7.02E-08
	Simple mode	23	0.02 [0.008, 0.031]	1.02 [1.008, 1.032]	2.52E-03
	Weighted mode	23	0.01 [0.001, 0.018]	1.01 [1.001, 1.018]	3.12E-02
Smoking	MR Egger	7	0.006 [-0.093, 0.106]	1.006 [0.911, 1.111]	9.05E-01
	Weighted median	7	0.035 [0.007, 0.064]	1.036 [1.007, 1.066]	1.34E-02
	Inverse variance weighted	7	0.036 [0.011, 0.06]	1.037 [1.011, 1.062]	4.20E-03
	Simple mode	7	0.06 [0.003, 0.117]	1.062 [1.003, 1.124]	8.38E-02
	Weighted mode	7	0.008 [-0.043, 0.059]	1.008 [0.958, 1.061]	7.70E-01
